# Biological Control of *Aspergillus flavus* by the Yeast *Aureobasidium pullulans* In Vitro and on Tomato Fruit

**DOI:** 10.3390/plants12020236

**Published:** 2023-01-04

**Authors:** Izabela Podgórska-Kryszczuk

**Affiliations:** Department of Analysis and Food Quality Assessment, University of Life Sciences in Lublin, Skromna 8, 20-704 Lublin, Poland; izabela.podgorska-kryszczuk@up.lublin.pl

**Keywords:** *Aureobasidium pullulans*, *Aspergillus flavus*, biocontrol, antagonistic yeast, fungal control

## Abstract

*Aspergillus flavus* is an important pathogenic fungus affecting many crops and is one of the main sources of their aflatoxin contamination. The primary method of limiting this pathogen is using chemical fungicides, but researchers focus on searching for other effective agents for its control due to many disadvantages and limitations of these agrochemicals. The results obtained in the present study indicate the high potential of two yeast strains, *Aureobasidium pullulans* PP4 and *A. pullulans* ZD1, in the biological control of *A. flavus.* Under in vitro conditions, mycelial growth was reduced by 53.61% and 63.05%, and spore germination was inhibited by 68.97% and 79.66% by ZD1 and PP4 strains, respectively. Both strains produced the lytic enzymes chitinase and β-1,3-glucanase after 5 days of cultivation with cell wall preparations (CWP) of *A. flavus* in the medium as a carbon source. In addition, the tested yeasts showed the ability to grow over a wide range of temperatures (4–30 °C), pH (4–11), and salinity (0–12%) and showed tolerance to fungicides at concentrations corresponding to field conditions. Both isolates tested were highly tolerant to cupric oxychloride, showing biomass gains of 85.84% (ZD1) and 87.25% (PP4). Biomass growth in the presence of fungicides azoxystrobin was 78.71% (ZD1) and 82.65% (PP4), while in the presence of difenoconazole, it was 70.09% (ZD1) and 75.25% (PP4). The yeast strains were also tested for antagonistic effects against *A. flavus* directly on tomato fruit. Both isolates acted effectively by reducing lesion diameter from 29.13 mm (control) to 8.04 mm (PP4) and 8.83 mm (ZD1).

## 1. Introduction

Food spoilage is a serious problem, leading to food waste and economic losses for producers and consumers. Microbial contamination, mainly by fungi, plays an essential role in this aspect. These microorganisms can occur at various stages of the food chain, such as harvesting, processing, and storing raw materials. Fungal growth leads to sensory changes in food but can also negatively impact health through mycotoxin production [1]. Fungi of the genus *Aspergillus* are widespread and particularly dangerous to humans and animals. Various species are opportunistic pathogens that can cause aspergillosis and respiratory allergies and can also cause aflatoxicosis due to the production of aflatoxins in food products. These mycotoxins are mainly produced by *A. flavus*, *A. parasiticus*, and *A. nomius* [2]. *A. flavus* is especially problematic because of its ability to grow on various substrates under different environmental conditions and the property to produce numerous spores that remain viable even under extreme conditions [3]. The entry of fungi into crops not only impairs the self-defense of crops by the fungal attack but also contaminates crop seeds, leading to the production of mycotoxins. Fungal infections affect crop growth and yield and cause market value loss [4]. *A. flavus* mainly contaminate corn, soybeans, peanuts, pistachios, Brazil nuts, cotton seeds, sunflower seeds, and rice [3,5]. Recently, the presence of these fungi has also been reported in fruits and vegetables, including strawberries [6], grapes [7], and tomatoes [8,9].

Tomato (*Solanum lycopersicum* L.) is an important commercial crop worldwide. The crop is cultivated for various purposes, such as using it for direct consumption and several industrial purposes in preparing tomato-based products [10]. Unfortunately, tomato is attacked by numerous pathogens, including fungi from the genera *Rhizoctonia*, *Alternaria, Aspergillus*, *Colletotrichum*, *Pythium*, and *Fusarium* [11]. The primary method of controlling pathogenic fungi and associated post-harvest diseases is synthetic chemical fungicides. However, their use is associated with drawbacks concerning handling hazards, awareness of fungicide residues in food, and risks to human health and the environment [8]. Therefore, in recent years there has been increased interest in biological methods of controlling fungal contamination in the field, including antagonistic microorganisms. The use of microorganisms as biological crop protection is not a new concept, but the growing interest in this method has resulted in the worldwide intensification of research into new solutions and the search for new effective strains. One group of antagonistic microorganisms used in biocontrol that is receiving increasing interest from scientists and industry is yeast. These microorganisms deserve special attention because they have simple nutritional requirements, do not produce allergenic spores or mycotoxins as many filamentous fungi do, and can grow rapidly and colonize a wide range of substrates under different conditions for long periods. The yeasts are found on the above-ground and underground parts of plants and use various mechanisms to effectively prevent the growth of pathogenic fungi during both the growing and storage periods [12,13].

*Aureobasidium pullulans* is a yeast-like fungus with high morphological and genetic diversity, found in most phyllosphere habitats and frequently isolated from the phyllosphere of fruits and vegetables [14]. This observed abundance has led many scientists to explore the potential of biocontrol for several plant pathogens, such as *Botrytis cinerea*, *Alternaria alternata* [15], *Penicillium expansum*, *Penicillium digitatum* [16], *Rhizoctonia solani* [17], and *Monilinia laxa* [18]. In addition, *A. pullulans* also produce pullulan, a water-soluble exopolysaccharide used in the food industry to form edible films. This kind of edible pullulan coating (with the addition of ethanolic propolis extract) obtained in a study by Pobiega et al. inhibited microbial growth on cherry tomatoes [19].

This study aimed to investigate the effectiveness of two new environmental yeast strains *A. pullulans* PP4 and *A. pullulans* ZD1 as biocontrol agents of the *A. flavus* in vitro and in vivo on tomato fruit.

## 2. Results

### 2.1. Confrontation Assay

**Figure 1:**
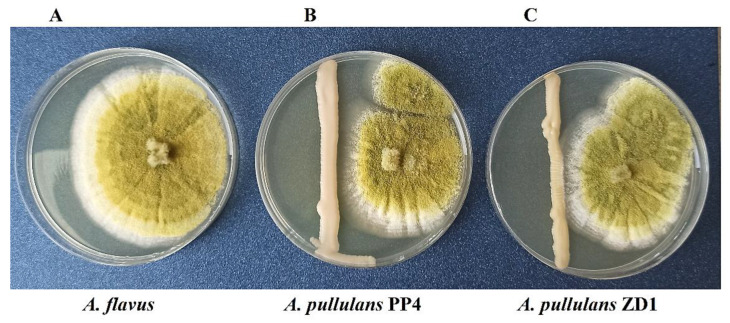
Growth inhibition of *A. flavus* caused by two yeast strains *A. pullulans* PP4 and *A. pullulans* ZD1 after 7 days of incubation at 28 °C on malt extract agar; (**A**)—*A. flavus* as a control; (**B**)—a dual culture of *A. flavus* and *A. pullulans* PP4; and (**C**)—a dual culture of *A. flavus* and *A. pullulans* ZD1.

The tested yeast strains *A. pullulans* PP4 and *A. pullulans* ZD1 reduced the growth of *A. flavus* on Petri dishes after 7 days of incubation (Figure 1, Table 1). The more effective strain in limiting the mycelial growth of *A. flavus* was the yeast *A. pullulans* PP4, inhibiting the growth of the pathogen by 63.05 ± 0.06%. The yeast *A. pullulans* ZD1 inhibited mycelial growth at 53.61 ± 0.06%.

### 2.2. Effect on A. flavus Spore Germination In Vitro

**Figure 2:**
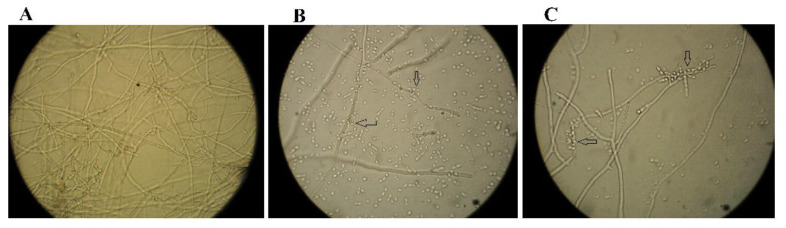
Effect of the yeasts *A. pullulans* PP4 and *A. pullulans* ZD1 on *A. flavus* spore germination; (**A**)—*A. flavus* as a control; (**B**)—*A. pullulans* PP4 with *A. flavus;* and (**C**)—*A. pullulans* ZD1 with *A. flavus*. Arrows indicate the accumulation of yeast cells at the mycelium of *A. flavus*.

The yeast tested in the study effectively inhibited the germination of *A. flavus* spores compared to the control after 24 h of incubation in a wort broth medium at 28 °C (Table 1). Similar to the confrontation assay, the more effective strain was the yeast *A. pullulans* PP4 limiting pathogen spore germination by 79.66 ± 1.15%. The strain *A. pullulans* ZD1 reduced the germination of *A. flavus* spores by 68.97 ± 1.0%. Under a light microscope, areas of yeast cell accumulation were observed at the mycelia of *A. flavus* (Figure 2).

### 2.3. Enzymatic Activities

**Figure 3:**
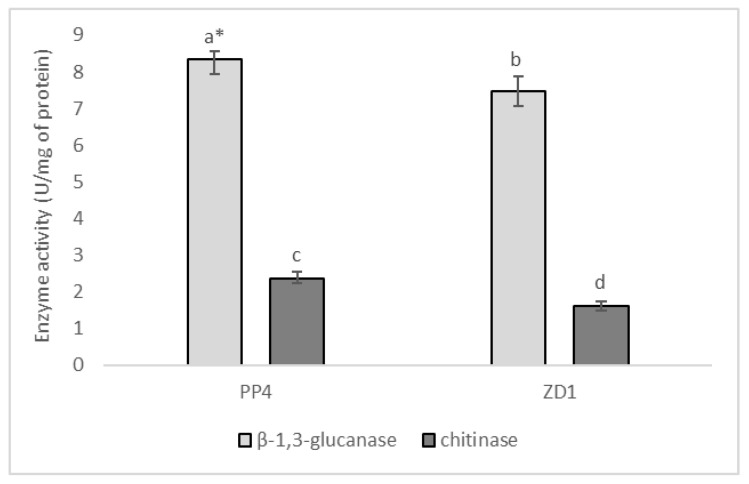
The activity of β-1,3-glucanase and chitinase on the 5th day of *A. pullulans* PP4 and *A. pullulans* ZD1 cultivation with *A. flavus* cell wall preparations in the medium as a carbon source. * values marked with the same letters do not differ significantly at *p* < 0.05 (Tukey’s post hoc test); error bars represent the standard deviation.

The tested yeasts *A. pullulans* PP4 and *A. pullulans* ZD1 produced the lytic enzymes chitinase and β-1,3-glucanase after 5 days of cultivation with the cell wall preparations (CWP) of *A. flavus* in the medium as a carbon source (Figure 3). In addition, both strains produced higher amounts of β-1,3-glucanase compared to chitinase. *A. pullulans* PP4 was found to be a better producer of both enzymes (8.35 ± 0.22 U/mg protein for β-1,3-glucanase and 2.37 ± 0.16 U/mg protein for chitinase) compared to the strain *A. pullulans* ZD1 (7.47 ± 0.42 U/mg protein for β-1,3-glucanase and 1.62 ± 0.13 U/mg protein for chitinase).

### 2.4. The Capacity of Yeast to Survive under Harsh Environmental Conditions

The results indicate that the yeast strains *A. pullulans* PP4 and *A. pullulans* ZD1 show the ability to grow over a wide range of temperatures, pH, and salinity. Both strains demonstrated active growth in the temperature range of 4–30 °C and a pH gradient of 4–11, maintaining an unchanged colony appearance compared to the control. There was no observed growth of the tested yeasts at 37 °C. Under saline conditions, the yeast *A. pullulans* ZD1 grew up to 10% NaCl, while *A. pullulans* PP4 grew up to 12% NaCl in the medium. However, the colony morphology of both strains changed under the influence of NaCl, becoming smaller by increasing the concentration of NaCl in the culture medium.

### 2.5. Effect of Fungicides on Tested Microorganisms In Vitro

**Figure 4:**
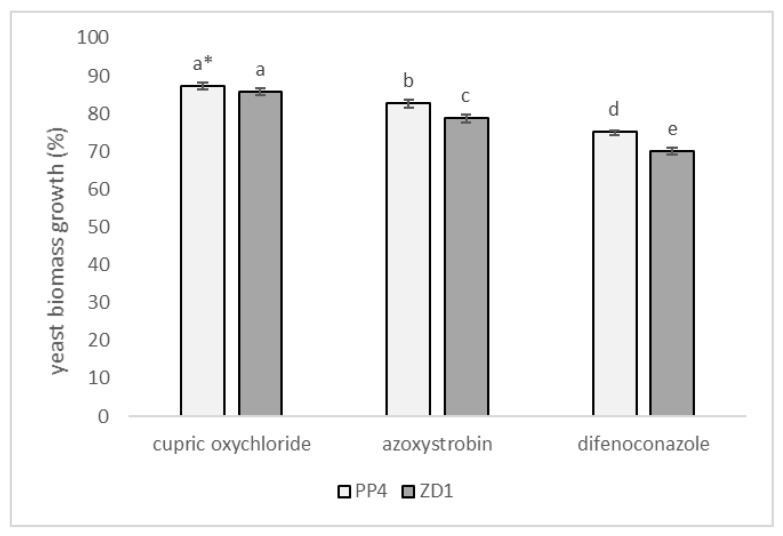
The influence of fungicides on the biomass production of the yeasts *A. pullulans* PP4 and *A. pullulans* ZD1; The maximum biomass production (100% growth) was taken as the biomass yield of the control trials; * values marked with the same letters do not differ significantly at *p* < 0.05 (Tukey’s post hoc test); and error bars represent the standard deviation.

The effect of fungicides with the active ingredients cupric oxychloride (0.5%), azoxystrobin (0.05%) and difenoconazole (0.02%) on the biomass production of the yeasts *A. pullulans* PP4 and *A. pullulans* ZD1 is shown in Figure 4. In general, the yeast *A. pullulans* PP4 proved more tolerant to the fungicides tested. However, the differences between isolates were not always statistically significant, as in the case of cupric oxychloride. Both tested isolates were the least sensitive to cupric oxychloride, showing a biomass growth of 87.25 ± 0.84% (strain PP4) to 85.84 ± 0.72% (strain ZD1) compared to the control. To azoxystrobin, the tested strains PP4 and ZD1 were moderately sensitive, showing a biomass yield of 82.65 ± 0.95% and 78.71 ± 1.01%, respectively. Of the fungicides studied, yeast was the most sensitive to difenoconazole, demonstrating a biomass growth of 75.25 ± 0.95% (PP4) to 70.09 ± 0.82% (ZD1) over the control.

### 2.6. Biocontrol of A. flavus by Yeast in Tomato Fruit

**Figure 5:**
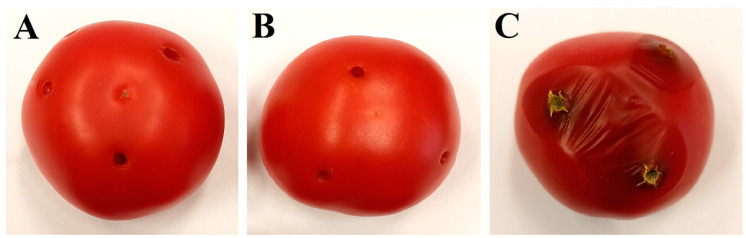

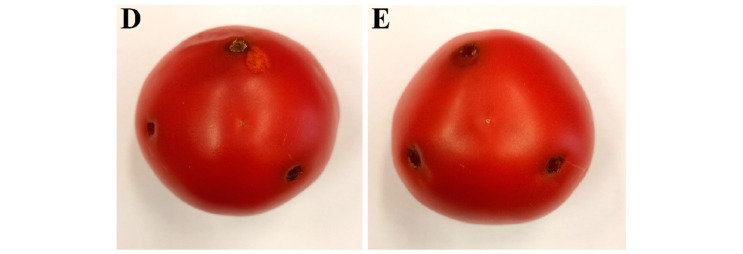
Biocontrol of *A. flavus* by yeasts *A. pullulans* PP4 and *A. pullulans* ZD1 in tomato fruit after 7 days of incubation at 21–22 °C. (**A**)—tomato inoculated with *A. pullulans* PP4 and sterile distilled water; (**B**)—tomato inoculated with *A. pullulans* ZD1 and sterile distilled water; (**C**)—tomato inoculated with *A. flavus* and sterile distilled water; (**D**)—tomato inoculated with *A. flavus* and *A. pullulans* PP4; and (**E**)—tomato inoculated with *A. flavus* and *A. pullulans* ZD1.

The potential of the yeasts *A. pullulans* PP4 and *A. pullulans* ZD1 to control *A. flavus* on tomato fruit was tested. No signs of microbial spoilage were observed on tomatoes treated only with the tested yeasts after 7 days (Figure 5A,B). After the same time, significant changes and evidence of spoilage were observed on fruit inoculated with the fungus *A. flavus* (Figure 5C). The tomatoes showed various signs of deterioration, such as the softening of the tissue around the wound, skin rupture with continuous liquid exudation, and the growth of external mycelium. The diameter of the lesions caused by the pathogen on the control fruit was 29.13 ± 2.15 mm. The tomatoes inoculated with the fungus *A. flavus* and the antagonistic yeasts *A. pullulans* PP4 and *A. pullulans* ZD1 maintained good condition after 7 days (Figure 5D,E). Only directly at the wound site were small lesions, and their diameters, when using the strains PP4 and ZD1 were 8.04 ± 1.37 mm and 8.83 ± 1.17 mm, respectively.

## 3. Discussion

Agricultural production requires the management of plant diseases to minimize crop losses, as well as to maintain crop quality. Due to the global importance of tomatoes as a fresh and processed product, there is great interest in improving disease-free tomato crops. However, in recent years, public concern about environmental pollution and problems with the development of pathogen resistance to fungicides has prompted a reduction in the use of chemical plant protection products. The current trend is to reduce the dose of fungicides and keep the number of applications to a minimum per season. In order to achieve such a purpose while maintaining adequate efficacy, a good way is to combine chemical fungicides with a biocontrol agent. The microbial biocontrol agent approach to tomato disease management is ecologically safe and capable of hindering or defeating pathogen populations [10,15,20]. Among the microorganisms antagonistic to plant pathogens, yeast meets many conditions for an effective biocontrol agent. Primarily, they can rapidly colonize plant surfaces, and many species can utilize nutrients from a variety of sources and can survive over a relatively wide range of temperatures and unfavorable conditions [21].

Antagonistic microorganisms, including yeast, exhibit many mechanisms of action, and competition for nutrients and space is considered one of the main ones, as it involves the nutritional requirements of both the antagonist and the pathogen. The investigation of competition for nutrients is relatively complicated because direct contact between the antagonist and the pathogen is not required, in contrast to competition for space. The two types of competition are commonly considered together without assigning each a corresponding significance level. The present study used a co-culture test of the yeast isolates *A. pullulans* PP4 and *A. pullulans* ZD1 with pathogenic fungi to select *A. flavus* growth inhibitors. Both tested isolates showed an effective reduction in mycelial growth (above 50%) on Petri dishes, confirming effective competition for space or nutrients against the phytopathogen. The yeast *A. pullulans* has been extensively studied by many scientists for its antagonistic effects against fungal pathogens and have proven efficacy towards many of them. Bencheqroun et al. [22] *in vitro* and *in situ* studies have shown that competition for apple nutrients, especially for amino acids, might be the primary mechanism for the activity of the *A. pullulans* Ach1-1 strain against blue mold caused by *P. expansum* on harvested apple fruit. The results obtained by Zhang et al. [23] also indicate that competition for nutrients played a significant role in the biocontrol activity of *A. pullulans* PL5 against *M. laxa, B. cinerea*, and *P. expansum*. The ability of *A. pullulans* PL5 to inhibit the growth of pathogen mycelium was significantly better when co-cultured with pathogens on a medium with a lower nutrient concentration. There are also reports in the literature on the effective reduction in *A. flavus* growth by antagonistic yeasts [24,25,26]. Competition for nutrients and space when co-culturing *A. flavus* with eight strains of the yeast *Saccharomyces cerevisiae* can be found in a study by Abdel-Kareem et al. [27]. The yeast reduced the growth of the pathogen by up to 74%. Sukmawati et al. [28] showed that the yeast, *A. pullulans*, effectively reduced the growth of *A. flavus* and *A. niger* by reducing the diameter of pathogen colonies on Petri dishes.

The ability of yeast to inhibit the germination of fungal spores can be very important for controlling these pathogens in the soil and under post-harvest conditions [29]. The literature shows that the ability of yeast to reduce spore germination depends on various mechanisms. Competition for nutrients directly impacts spore germination because conidia require nutrients (carbon sources, nitrogen, glucose, fructose, and iron). However, other factors, such as volatile organic compounds, enzymes, and antibiotic-like compounds, play an essential role in this process [16]. Hua et al. [30] showed that 2-phenylethanol, a volatile organic compound produced by the yeast *Pichia anomala*, inhibited spore germination, mycelial growth, and even aflatoxin production by the fungus *A. flavus.* The alkaline serine protease *ALP5* from *A. pullulans* reduced the spore germination and spore tube length of *P. expansum*, *B. cinerea*, *A. alternata,* and *Monilinia fructicola* in vitro and showed a concentration-dependent inhibitory effect against these pathogens on apple [31,32].

Cell walls of filamentous fungi consist of 50–60% glucan, 20% or more chitin, and about 20–30% protein in the dry weight of the wall [16]; therefore, the production of lytic enzymes, including chitinase and β-1,3-glucanase, is an important mechanism of yeast antagonistic action against pathogenic fungi. It has been suggested that extensive production of extracellular lytic enzymes by yeast may play an important role by enhancing nutrient competition or by some other unknown mechanisms [23]. Various factors influence microbial enzyme production, including strain, culture conditions, and duration. The type of culture medium used (including carbon sources) is also important, as the medium provides nutrients for the microorganisms. The use of different culture media often provides different results [33]. In the studies presented in this paper, the inducer of enzyme production was CWP of *A. flavus* as a carbon source in the medium. Both tested strains of *A. pullulans* produced chitinase and β-1,3-glucanase, a property confirmed in the literature for this species [16,23].

Many polyextremotolerance fungi (demonstrating significant stress tolerance) are ubiquitous in the environment and are often found on plant surfaces. Therefore, several have been proposed and used as biocontrol agents in agriculture [34]. *A. pullulans* is one of the most common inhabitants of the phyllosphere of many crop plants and is also found in many other habitats [35]. These yeasts can also thrive in various environmental conditions (e.g., hypersaline habitats, arid conditions, glaciers, and radiation sites) due to the presence of genes involved in stress tolerance [20]. The presented study examined the various aspects of polyextremotolerance of the tested strains of *A. pullulans*, including tolerance to low and high temperatures, pH, and salinity. The isolates of *A. pullulans* PP4 and *A. pullulans* ZD1 showed active growth at 4–30 °C, which is desirable for biocontrol agents. Temperature regimes on the surface of fruit and leaves in the field can vary widely—high temperatures during the day and slightly lower temperatures at night. Therefore, microorganisms antagonistic to plant pathogens should be adapted to such situations. The yeasts tested were capable of active growth even under refrigeration conditions of 4 °C. This is an important biocontrol trait, as many vegetables and fruits are stored under such conditions. The growth of the tested isolates was inhibited entirely at 32 °C and above, eliminating concerns about the pathogenicity of *A. pullulans* in humans. The ability to grow at 37 °C and above is the most obvious virulence factor for human fungal pathogens and one of the most important risk factors when evaluating the safety of potential biocontrol agents [34]. Both tested yeast strains *A. pullulans* PP4 and *A. pullulans* ZD1 actively grew in a pH gradient of 4–11, consistent with the literature data for this species [36]. This is an essential feature because environmental conditions (including pH and temperature) affect the biosynthesis of various compounds by *A. pullulans* [37,38]. Depending on the strain, the yeast *A. pullulans* can tolerate different concentrations of NaCl. Zajc et al. [34] showed that this species could grow even at 18% NaCl in the culture medium. In the study presented in this paper, active yeast growth was demonstrated at 10% and 12% NaCl concentrations for *A. pullulans* ZD1 and *A. pullulans* PP4, respectively. By increasing the concentration of NaCl in the culture medium, the morphology of the colonies became smaller, which was also confirmed in other studies [36]. Data from the literature suggest that tolerance to high osmolarity may be related to adaptation to other stress conditions, such as oxidative stress, heat and cold stress, and heavy-metal-induced stress [39], which may also play an essential role in biocontrol. The tolerance of the yeast *A. pullulans* to different ecological conditions allows them to colonize many niches and guarantees adaptability and survival [36].

The effective antagonist acts against pathogens comparably to chemical pesticides, as shown in a study by Dimakopoulou et al. [40]. In their experiment, *A. pullulans* Y-1 was as effective as the commercial fungicide fludioxonil + cyprodinil in reducing *Aspergillus carbonarius* on berries. Fungicides, commonly used in conventional crop protection, mainly act on basic fungal functions such as respiration, sterol biosynthesis, or cell division [41]. However, when these compounds are used to protect plants from pathogens, they can also affect non-target microorganisms found in the plant’s phyllosphere. Since these microorganisms also often have great potential to inhibit pathogen growth, the effects of fungicides on non-target saprotrophic fungi should be investigated. Under field conditions, these effects are difficult to monitor because saprotrophs are affected by many factors, including the growth stage, weather, and the health status of the protected plants [42]. In vitro studies provide valuable information on the sensitivity of different yeast species to pesticides. Therefore, the present study examined the tolerance of the yeasts *A. pullulans* PP4 and *A. pullulans* ZD1 to fungicides commonly used in tomato protection: cupric oxychloride, azoxystrobin, and difenoconazole. Both strains tested were highly tolerant to the investigated agrochemicals, showing a biomass growth of 87.25–75.25% (strain PP4) and 85.84–70.09% (strain ZD1) compared to the control. The results obtained in the present study confirm the tolerance of the yeast *A. pullulans* to selected fungicides agree with reports from the literature. Vadkertiová and Sláviková [41] showed a high tolerance of *A. pullulans* yeast isolated from fruit tree leaves to several different fungicides. In the presence of an agent with the active ingredient cupric oxychloride, the biomass yield of these yeasts was 80% compared to the control. Wachowska et al. [43], studying the effects of several different fungicides on yeast populations colonizing wheat, included azoxystrobin among the least toxic agrochemicals. In their study, 86–100% of tested yeast species isolates were not sensitive to azoxystrobin. The authors observed a mutation in the *CYTb* gene (G143A) in the azoxystrobin-resistant yeast A. *pullulans*. Magoye et al. [20] showed that the baseline minimum inhibitory concentration (MIC50) values determined for 30 different strains of *A. pullulans* and 3 fungicides, such as captan, cyprodinil, and difenoconazole, were higher than the concentrations of the respective fungicides used under field conditions to control plant pathogens.

The susceptibility of the test microorganism to fungicides is influenced by many factors, including its original location. It was proven in a study by Vadkertiová and Sláviková [41], showing that yeast isolated from forest trees was more sensitive to pesticides than yeast isolated from fruit trees previously treated with pesticides. In the study presented in this paper, yeast was also isolated from fields where crop protection products, including fungicides, were applied during the season. We suspect this may have significantly affected the tolerance of the strains *A. pullulans* PP4 and *A. pullulans* ZD1 against the tested agrochemicals. Magoye et al. [20] suggest that this yeast exhibits a rather pleiotropic mechanism of tolerance to fungicides instead of a specific resistance mechanism to a particular agent. The study presented in this paper and a report by other authors [20] indicate that high abundance in the environment, competitiveness, and stress tolerance are associated with low sensitivity to antifungal agents. The unique genetic and biochemical properties that make *A. pullulans* particularly tolerant to stress may also confer nonspecific insensitivity to antifungal compounds [44]. Intrinsic insensitivity to fungicides in antagonistic yeasts intended for use as biocontrol agents can be precious [20]. Antagonistic microorganisms combined with a fungicide would allow more effective and safer consumer protection of crops [40].

The present study tested the potential of the yeasts *A. pullulans* PP4 and *A. pullulans* ZD1 to control *A. flavus* directly on tomato fruit. The studied isolates were characterized by high efficiency against the pathogen, preventing the spread of infection. Yeast generally can efficiently assimilate a wide range of mono- and disaccharides, making these nutrients unavailable to pathogens and allowing yeast to reproduce rapidly. It is especially important for wound pathogens, which usually depend on exogenous nutrients for their growth [18,45]. In addition, to compete effectively with the pathogen, the antagonistic microorganism must quickly adapt to the host’s different environmental and nutritional conditions [18]. *A. pullulans* has been shown to produce a variety of enzymes, polymers, lipids, volatile compounds, and secondary metabolites that give it specific antagonistic activity against bacteria and fungi [12]. Other researchers also demonstrated the potential of *A. pullulans* as biocontrol agents in tomato pathogen control. A study by Di Francesco et al. [46] proved that the yeast strain *A. pullulans* AP3 reduced the incidence of *B. cinerea* in tomatoes by 80.9%. In their study, Shi et al. [47] proved that *A. pullulans* S2 yeast inhibited the occurrence of rot, maintained fruit firmness, and reduced tomato weight loss. The researchers also showed that the pre-harvest application of *A. pullulans* S2 remodeled the tomato surface microbiome leading to changes in the bacterial and fungal community that can inhibit plant pathogens and reduce fruit disease incidence [48].

## 4. Materials and Methods

### 4.1. Microorganisms

The fungi and yeasts used in the experiment were isolated from the environment—*A. flavus* from soil sampled from a vegetable garden and two yeast strains *A. pullulans* PP4 and *A. pullulans* ZD1, from ears of wheat from two different fields located in eastern Poland. The microorganisms were genetically identified according to the methodology previously described [33], using the universal primers ITS1 (50-TCCGTAGGTGAACCTGCGG-30) and ITS4 (50-TCCTCCGCTTATTGATATGC-30), and deposited in the Department of Analysis and Food Quality Assessment of the University of Life Sciences in Lublin, Poland. The pure cultures of fungi and yeasts were kept fresh and viable by periodical transfers on malt extract agar medium (30 g malt extract, 5 g mycological peptone, 15 g agar per 1 L of distilled water; pH 5.5 ± 0.2) under aseptic conditions throughout the study. Strains were stored at 4 °C for routine cultivation.

### 4.2. Confrontation Assay

Inhibition of *A. flavus* mycelial growth by the tested yeasts was assessed in Petri dishes containing 20 mL malt extract agar. In the confrontation test, a disc of pathogen mycelium (5 mm in diameter) cut from a 7-day-old culture was placed at about 3 cm from one edge of the dish. Then, it was inoculated by linear streaking with a loop of 2-day-old yeast culture at about 3 cm from the opposite edge of the plate. The control was a Petri dish inoculated with only the fungal pathogen. Three replicates were performed for each treatment. After 7 days at 28 °C, the radius of *A. flavus* mycelium in the direction of yeast (R2) and the radius of mycelium in the control sample (R1) were measured. Percentage inhibition of pathogen growth was calculated according to the formula [49]:

Inhibitory activity (%) = (R1 − R2)/R1 × 100

### 4.3. Effect on A. flavus Spore Germination In Vitro

The effect of the tested yeasts on *A. flavus* spore germination was assessed in wort broth medium (15 g malt extract, 1 g peptone, 12.5 g maltose, 2.5 g glucose, 1 g K_2_HPO_4_, and 1 g NH_4_Cl per 1 L of distilled water; pH 4.8 ± 0.2). Yeast cells were grown at 28 °C for 48 h in YPG medium (20 g glucose, 20 g peptone, 10 g yeast extract per 1 L of distilled water; pH 6.0 ± 0.2) and were harvested by centrifugation at 10,000 rpm for 10 min and then resuspended in sterile Ringer’s solution. Next, 100 µL of living cells of the yeasts (5 × 10^8^ cells per mL) and 100 µL of a 10-day-old *A. flavus* culture spore suspension (5 × 10^6^ spores per mL) in Ringer’s solution were transferred to tubes containing 4.8 mL wort broth medium. As a control, 100 µL of pathogen spore suspension was added to a 4.9 mL medium. Then, the tubes were incubated at 28 °C on a rotary shaker at 150 rpm for 24 h. After this time, in vivo preparations were made by applying a drop of the co-culture to a Thoma counting chamber. The number of germinated spores per one hundred observed was counted in three replicates.

### 4.4. Enzymatic Activities

Yeasts were cultured in a medium containing 3 g yeast extract, 5 g (NH_4_)_2_SO_4_, 5 g KH_2_PO_4_ supplemented with 5 g of *A. flavus* CWP per 1 L of distilled water. CWP were prepared according to the methodology reported by Chan and Tian [50]. An amount of 30 mL of culture medium was added to 100 mL flasks and inoculated with 0.5 mL of yeast suspension (5 × 10^8^ cells per mL). The flasks were incubated at 28 °C at 150 rpm for 5 days. Culture filtrates were taken from each flask and centrifuged at 10,000 rpm for 15 min. The supernatants were used to analyze chitinase, β-1,3-glucanase, and protein contents according to the previously described methodology [33]. Chitinase and β-1,3-glucanase activities were assessed by measuring the reducing sugars released from colloidal chitin and laminarin (Sigma-Aldrich, Poznań, Poland), respectively. One unit (U) of each enzyme activity was defined as the amount of the enzyme that yielded 1 μmol of glucose equivalent per milligram of protein per minute. Each assay was performed in triplicate.

### 4.5. The Capacity of Yeasts to Survive under Harsh Environmental Conditions

The ability of yeast to survive in harsh environmental conditions was analyzed in Petri dishes for three characteristics: temperature, pH, and salinity. In each test, 100 µL of live yeast cells (5 × 10^8^ cells per mL) were inoculated into a YPD medium (10 g yeast extract, 20 g peptone, 20 g glucose, 20 g agar per 1 L distilled water; pH 6.0 ± 0.2) and incubated for 72 h. To assess the effect of temperature on yeast growth, they were incubated at 4, 14, 20, 24, 28, 30, 32, 35, and 37 °C. To determine the effect of pH on the growth of the tested yeasts, tests were carried out at pH 4, 5, 6, 7, 9, and 11 at 28 °C. Different salt concentrations were used to determine the effect of salinity: 0, 2, 4, 6, 8, 10, 12, and 14% NaCl, and the dishes were incubated at 28 °C. Each experiment was carried out in triplicate.

### 4.6. Effect of Fungicides on Tested Yeast In Vitro

The impact of the selected fungicides on the growth of tested yeast strains *A. pullulans* PP4 and *A. pullulans* ZD1 was tested according to the methodology provided by Vadkertiová and Sláviková [41] with slight modifications. In the study, 3 different fungicides recommended for tomato protection were used, containing the following active substances: cupric oxychloride, azoxystrobin, and difenoconazole. In this assay, 0.5 mL of yeast inoculum (5 × 10^8^ cells per mL) was added to tubes containing 9.5 mL of sterile YPG medium with the fungicide addition in the concentration of active ingredient recommended by the manufacturer for field treatments: cupric oxychloride 0.5%, azoxystrobin 0.05%, difenoconazole 0.02%. The control sample was a tube without fungicide inoculated in the same way. The samples were incubated at 28 °C on a rotary shaker at 150 rpm for 7 days. After this time, the biomass yields were measured and compared with the biomass production in control samples. Yeast cultures were centrifuged at 5000 rpm at 4 °C for 10 min, and the supernatant was decanted. The growth yield of the yeasts was determined as dry biomass (drying at 105 °C to constant weight). The experiment was performed in triplicate.

### 4.7. Biocontrol of A. flavus by Yeast in Tomato Fruit

The effectiveness of *A. flavus* biocontrol by yeast *A. pullulans* on tomato fruit cv. Krakus purchased from a local store (Lublin, Poland) was evaluated. Tomato fruits were washed in warm water, air-dried, and externally disinfected using 80% ethanol. Three wounds were made on each fruit using a sterile pipette tip (5 mm in diameter), and 30 μL of yeast suspension (5 × 10^8^ cells per mL) in sterile distilled water was applied to each. Next, 2 h later, each wound was inoculated with 15 μL of *A. flavus* spore suspension (5 × 10^6^ spore/mL). Two controls were performed: (1) fruit inoculated with fungus *A. flavus* (15 μL; 5 × 10^6^ spore/mL) and 30 μL sterile distilled water; (2) fruit inoculated with yeast (30 μL; 5 × 10^8^ cells per mL) and 15 μL sterile distilled water. Treated tomatoes were stored at room temperature (21–22 °C) in closed, sterile plastic containers for 7 days. The diameter of lesions on the fruit caused by *A. flavus* was determined. Eight tomatoes were used for each trial, and the experiment was performed in triplicate.

### 4.8. Statistical Analysis

The results were analyzed statistically using Statistica 13.3 (Statsoft, Cracow, Poland) and Excel 2019 (Microsoft, Washington, DC, USA). An analysis of variance (ANOVA) was carried out to compare the results, and the significance of differences between group means was determined using Tukey’s post hoc test. All statistical hypotheses were verified at the significance level of *p* < 0.05.

## 5. Conclusions

The results indicate the high potential of the yeasts *A. pullulans* PP4 and *A. pullulans* ZD1 in controlling *A. flavus*, a dangerous pathogen of many crop plants. Both strains effectively reduced spore germination, produced the lytic enzymes chitinase and β-1,3-glucanase, inhibited mycelial growth in vitro, and prevented the pathogen from spreading on tomato fruit. The ability of the tested yeasts to grow under different conditions of temperature, pH, and salinity may allow them to colonize many niches and guarantee adaptability and survival. High yeast tolerance to selected fungicides, such as cupric oxychloride, azoxystrobin, and difenoconazole, is a precondition for possible combinations and synergistic action of biocontrol agents and fungicides.

## Figures and Tables

**Table 1:** In vitro effects of tested yeast on *A. flavus*.

Yeast	Inhibition (%)	
	Mycelial Growth	Spore Germination
*A. pullulans* PP4	63.05 ± 0.06 a *	79.66 ± 1.15 a
*A. pullulans* ZD1	53.61 ± 0.06 b	68.97 ± 1.0 b

* Within each column, values marked with the same letters do not differ significantly at *p* < 0.05 (Tukey’s post hoc test); ± standard deviation.

## Data Availability

The data presented in this study are available on request from the corresponding author.
